# Mixed methods evaluation of simulation-based training for postpartum hemorrhage management in Guatemala

**DOI:** 10.1186/s12884-022-04845-2

**Published:** 2022-06-24

**Authors:** Pooja S. Parameshwar, Katherine Bianco, Elizabeth B. Sherwin, Pamela K. Meza, Alisha Tolani, Paige Bates, Lillian Sie, Andrea Sofía López Enríquez, Diana E. Sanchez, Edgar R. Herrarte, Kay Daniels

**Affiliations:** 1 Stanford University Medical Center, Stanford, CA HH333 USA; 2 Department of Obstetrics & Gynecology, Division of Maternal-Fetal Medicine, Stanford University School of Medicine, Stanford, USA; 3 Hospital de Gineco Obstetricia, Instituto Guatemalteco de Seguridad Social - Universidad de San Carlos de Guatemala, Guatemala City, Guatemala

**Keywords:** Medical education, Obstetrics, Maternal mortality, Simulation-based training, Global health

## Abstract

**Background:**

To assess if simulation-based training (SBT) of B-Lynch suture and uterine balloon tamponade (UBT) for the management of postpartum hemorrhage (PPH) impacted provider attitudes, practice patterns, and patient management in Guatemala, using a mixed-methods approach.

**Methods:**

We conducted an in-country SBT course on the management of PPH in a governmental teaching hospital in Guatemala City, Guatemala. Participants were OB/GYN providers (*n* = 39) who had or had not received SBT before. Surveys and qualitative interviews evaluated provider knowledge and experiences with B-Lynch and UBT to treat PPH.

**Results:**

Multiple-choice surveys indicated that providers who received SBT were more comfortable performing and teaching B-Lynch compared to those who did not (*p* = 0.003 and 0.005). Qualitative interviews revealed increased provider comfort with B-Lynch compared to UBT and identified multiple barriers to uterine balloon tamponade implementation.

**Conclusions:**

Simulation-based training had a stronger impact on provider comfort with B-Lynch compared to uterine balloon tamponade. Qualitative interviews provided insight into the challenges that hinder uptake of uterine balloon tamponade, namely resource limitations and decision-making hierarchies. Capturing data through a mixed-methods approach allowed for more comprehensive program evaluation.

**Supplementary Information:**

The online version contains supplementary material available at 10.1186/s12884-022-04845-2.

## Background

Maternal mortality is a significant concern in the developing world, where the majority of all maternal deaths occur [1]. Most maternal deaths are preventable and disproportionately affect the poorest and youngest women worldwide. The 2016–2030 Sustainable Development Goals’ target for maternal mortality is to reduce the global maternal mortality ratio to less than 70 per 100,000 live birth by 2030 [2, 3]. Although there have been global reductions in maternal mortality in recent years, Latin America still faces challenges. In particular, Guatemala has one of the highest maternal mortality rates in Latin America, with a rate of 88 deaths per 100,000 live births [4] in 2015. Causes for maternal mortality in Guatemala include postpartum hemorrhage (PPH), hypertension, sepsis, and unsafe abortion [5].

Given that the high maternal mortality rate may in part be due to limited training of health workers to adequately identify, prevent, or handle such emergencies [6], healthcare worker training programs have grown in popularity. Simulation-based training (SBT) for educating health professionals is one model that has consistently been found to improve knowledge, skills, behaviors, and patient-related outcomes [4]. SBT can be especially helpful in low and middle income countries (LMICs) given that it does not require expensive resources beyond human capital to facilitate teaching. By participating in simulations, trainees practice medical skills, effective communication, and teamwork under pressure, which can enable them to provide better and safer care to patients. Given the high acuity of obstetric emergencies and need for team-based care, SBT has been used to train healthcare workers in the management of obstetric emergencies in an effort to reduce maternal mortality [7–11].

We and others have published studies [12] evaluating SBT in LMICs with a focus on short and longer-term retention of learning, however there is still a need to better understand the process of implementation and uptake of training into local practice cultures [13]. In order to better elucidate the long-term impact of SBT on obstetric emergencies, this study sought to provide an in-depth look at one institution’s experience implementing SBT for the management of postpartum hemorrhage (PPH) in Guatemala. Using a mixed-methods approach, we aimed to assess if the introduction of SBT impacted provider attitudes, practice patterns, or patient management.

## Methods

This study was approved by the Stanford University Institutional Review Board.

Study activities took place at the Instituto Guatemalteco de Seguridad Social (IGSS) Pamplona hospital in Guatemala City, Guatemala. Data collection was overseen by Stanford University and IGSS hospital faculty.

IGSS Pamplona is a large, tertiary academic hospital that manages approximately 8000 to 9000 deliveries per year. It is considered a high-risk hospital that receives referrals from neighboring hospitals for complicated pregnancies. IGSS Pamplona does not have an Adult Intensive Care Unit (ICU), however, and sends postpartum ICU patients to an affiliated, nearby sister site. IGSS Pamplona has PPH management protocols in place that are based on World Health Organization (WHO) and national guidelines.

The Global Outreach-Mobile Obstetrics and Medical Simulation (GO MOMS) program is a SBT program developed at Stanford University to provide a standardized obstetrical and gynecological training tool for faculty and resident learners within teaching hospitals in low-resource settings [14]. Topics covered in GO MOMS include B-Lynch, uterine balloon tamponade (UBT), management of pre-eclampsia/eclampsia, shoulder dystocia, placement of foley catheters for cervical ripening, and maternal cardiac arrest. A needs assessment was carried out prior to the first GO MOMS course in Guatemala. This revealed that PPH was a leading cause of morbidity, thus GO MOMS directed a large component of the SBT to addressing PPH management. Through SBT, GO MOMS teaches the techniques of B-Lynch suture and uterine balloon tamponade (UBT), recommended for the treatment of PPH by the WHO and others [15, 16]. For UBT, two techniques were taught using different devices: 1) Bakri balloons and 2) condom-catheter uterine balloon tamponades. Both devices were demonstrated given that access to Bakri balloons is limited in many places and condom-catheter UBTs may be more accessible but require additional steps for assembly.

### Study participants

The intervention group consisted of Guatemalan OB/GYN residents and/or attending physicians who had participated in the GO MOMS simulation program at least once prior to 2019 (and who were participating in GO MOMS training again in 2019) (*n* = 24). The comparison group consisted of Guatemalan OB/GYN residents or attending physicians who had not yet participated in the GO MOMS simulation program (and who were participating in the program for the first time in 2019) (*n* = 15). The 2019 GO MOMS simulation program was held at a large conference, and individuals could opt in or out of participating in our study after completion of the GO MOMS simulation training session.

Study information was provided to all participants. They were informed that we were conducting a study to help improve simulation training programs and evaluate experiences of participants. Verbal consent was obtained for all participants for both the multiple-choice knowledge survey and qualitative components of the study.

### Multiple-choice knowledge survey

A multiple-choice knowledge survey was designed to gather demographic information, practice patterns, prior training, knowledge, and experience with B-Lynch, UBT, and medical abortion (for the management of spontaneous abortion). The data on medical abortion will be published separately.

For the multiple-choice knowledge survey, we utilized convenience sampling by allowing all residents and attending physicians participating in the GO MOMS program in 2019 (some of who had also participated in the GO MOMS program prior to 2019) to participate in the study. Study participants completed the multiple-choice questionnaires via REDCap in September 2019. The Stanford REDCap platform (http://redcap.stanford.edu) is developed and operated by the Stanford Medicine Research IT team. Participants completed the RedCap survey using a laptop computer, iPad, smartphone, or via pen and paper. Study participants who completed the REDCap surveys could “opt-in” to also participate in qualitative interviews.

### Qualitative interview

A qualitative interview guide was designed to gather information concering challenges, positive experiences, and attitudes of providers around B-Lynch and UBT. The qualitative interview guide included question prompts and interviewer scripts.

A purposeful sampling strategy was utilized for the qualitative interviews. Our goal was not to generalize to a population with statistical confidence, but to select an information-rich population. This was deemed appropriate for our study as we sought to understand perspectives and in-depth points of view around provider experiences with implementation of B-Lynch and UBT for PPH beyond the multiple-choice knowledge survey alone [17]. We selected OB/GYN residents or attending physicians who had participated in GO MOMS training in the past (at least once prior to 2019) to gain more information about their experiences incorporating knowledge gained from GO MOMS into clinical practice.

All qualitative interviews were conducted using the qualitative interview guide via an interpreter fluent in Spanish with the support of a research assistant who speaks English. Interviews were audiotaped, translated into English, and transcribed by study personnel.

### Multiple-choice knowledge survey and qualitative interview

For both the multiple-choice knowledge survey and the qualitative interview guide, all questions were written in English and validated via peer review by U.S. board-certified OB/GYNs for accuracy. The questionnaires were translated into Spanish and reviewed by IGSS faculty. A back translation was performed to ensure that the questions asked in Spanish represented the same content and meaning as those in English.

### Data analysis

For the multiple-choice knowledge surveys, Fisher’s exact test was utilized with *p* < 0.05 considered statistically significant. Analyses were performed using SAS Enterprise Guide 7.1 (SAS Institute Inc., Cary, NC).

For the qualitative interviews, grounded theory methodology, as described by Charmaz [18], was utilized for data analysis. This method of qualitative analysis is constructivist, as it generates concepts and theories from data. Two independent investigators carried out the qualitative analysis by conducting line-by-line coding of interview transcripts. Preliminary themes were then developed by grouping similarly coded phrases. Emergent concepts were then derived by grouping preliminary themes into categories. One investigator was fluent in Spanish and English to ensure that any lack of clarity surrounding the translated content could be addressed. Investigators who participated in coding had prior experience in qualitative methods and conducting OB/GYN-related research.

## Results

### Multiple-choice survey

A total of 39 physicians participated in our study: 24 individuals in the intervention group and 15 individuals in the comparison group. Table 1 provides demographic information for all study participants who responded to the RedCap multiple-choice knowledge surveys. For the purposes of this study, only residents and attending physicians with at least 1–4 years of hospital practice were included in our analysis (*n* = 39). This meant that no first year residents were included, as they had less than one year of hospital practice.

**Table 1:** Demographics and practice patterns among survey respondents (*n* = 39)

Characteristics	Study Participants *n* = 39 (%)
Job Title	
2^nd^ Year Resident	13 (33.3)
3^rd^ Year Resident	10 (25.6)
4^th^ Year Resident	3 (7.7)
Attending	13 (33.3)
Vaginal deliveries performed per month	
0–10	18 (46.2)
10–30	8 (20.5)
> 30	12 (30.8)
Not sure	1 (2.6)
Cesareans performed per month	
0–10	9 (23.1)
10–30	14 (35.9)
> 30	14 (35.9)
Not sure	1 (2.6)
Postpartum hemorrhages (more than 1000 cc loss after delivery) per month	
0–10	36 (92.3)
10–30	1 (2.6)
Not sure	1 (2.6)
General Practice Patterns	
Previously performed B-lynch	*n* = 14 (%)
When you did a B-Lynch, how many of them were done at time of C-sections?	
All	9/14 (64.3)
Most	1/14 (7.1)
Some	1/14 (7.1)
Few	1/14 (7.1)
None	2/14 (14.3)
When you did a B-Lynch, how many of them were done after vaginal deliveries?	
All	1/14 (7.1)
Few	1/14 (7.1)
None	12/14 (85.7)
How often after performing a B-Lynch did you still have to proceed to hysterectomy to control the bleeding?	
Most of the time	3/14 (21.4)
Sometimes	2/14 (14.3)
A few times	4/14 (28.6)
Never	5/14 (35.7)
Previously performed UBT	*n* = 13 (%)
When you did a UBT, how many of them were done at time of C-sections?	
Some	2/13 (15.4)
Few	3/13 (23.1)
None	10/13 (76.9)
When you did a UBT, how many of them were done after vaginal deliveries?	
All	5/13 (38.5)
Most	2/13 (15.4)
Some	2/13 (15.4)
Few	2/13 (15.4)
None	3/13 (23.1)
How often after performing a UBT did you still have to proceed to hysterectomy to control the bleeding?	
Sometimes	6/13 (46.2)
A few times	3/13 (23.1)
Never	6/13 (46.2)

Table 2 compares where providers were first taught B-Lynch and UBT based on whether or not they had received previous GO MOMS training. Participants without GO MOMS training were significantly less likely to have ever been taught UBT and B-Lynch (*p* = 0.0353 and *p* = 0.0153, respectively). Specifically, participants with previous GO MOMS training were more likely to have been taught B-Lynch and UBT by the GO MOMS course or another resident or attending compared to those who had not received previous GO MOMS training (*p* = 0.0086 and 0.0068, respectively).

**Table 2:** Previous training experience with B-Lynch and UBT among survey respondents (*n* = 39)

	**Previous GO MOMS Training (%) (*n* = 24)**	**No Previous GO MOMS Training (%) (*n* = 15)**	***p*-value**^**1**^
Ever been taught B-Lynch	22 (91.7)	8 (53.3)	**0.0153**
First learned B-Lynch from:			**0.0086**
GO MOMS Course	7 (31.8)	0 (0.0)	
Another resident or attending	15 (65.2)	6 (75.0)	
Independent study (read about it)	0 (0.0)	2 (25.0)	
Ever been taught UBT	19 (78.2)	6 (40.0)	**0.0353**
First learned UBT from:			**0.0068**
GO MOMS Course	13 (68.4)	0 (0.0)	
Another resident or attending	3 (15.8)	4 (66.7)	
Independent study (read about it)	3 (15.8)	2 (33.3)	

^1^*p*-values calculated using Fisher’s Exact Test

Table 3 compares provider comfort with B-Lynch and UBT based on whether or not they had received previous GO MOMS training. Participants with previous GO MOMS training reported feeling more comfortable doing B-Lynch with and without supervision and were also more likely to have taught B-Lynch to someone else compared to those who had not received previous GO MOMS training (*p* = 0.0030 and 0.0049, respectively). However, there was no difference between those with and without previous GO MOMS training in terms of comfort with UBT or teaching UBT to others.

**Table 3:** Previous teaching experience and comfort with B-Lynch and UBT among survey respondents (*n* = 39)

	**Previous GO MOMS Training (%) (*n* = 24)**	**No Previous GO MOMS Training (%) (*n* = 15)**	***p*-value**^1^
Taught B-lynch to another	13 (54.2)	1 (6.7)	**0.0049**
Comfort with B-Lynch			**0.0030**
Can do without supervision	6 (25.0)	0 (0.0)	
Can do with supervision	13 (54.2)	2 (18.2)	
Not comfortable	5 (20.8)	9 (81.8)	
Taught UBT to another	9 (39.1)	4 (28.6)	0.7245
Comfort with UBT			0.1014
Can do without supervision	11 (45.8)	2 (14.3)	
Can do with supervision	7 (29.2)	4 (28.6)	
Not comfortable	6 (25.0)	8 (57.1)	

^1^*p*-values calculated using Fisher’s Exact Test

### Qualitative interviews

In total, 11 participants took part in the qualitative interviews. Eleven themes were generated from the qualitative analysis. Themes included hospital practice patterns and use of techniques (UBT and B-Lynch), hospital resource and personnel limitations, decision-making hierarchies, challenging nature of emergencies, and impact of SBT (Table 4).

**Table 4:** Themes and illustrative quotes pertaining to themes^a^

**Hospital Practice Patterns and Use of Techniques (UBT and B-Lynch)**		
Lack of practice and exposure to techniques (B-Lynch and UBT)	•B-Lynch: Lack of practice •B-Lynch: Not done routinely •UBT: Never used •UBT: Less frequent than B-Lynch	It’s the technique. We know the technique from our books, but in the moment, it’s difficult. We just don’t do it that often. So if we practice more, it won’t be as big of a deal to do it.
Importance of overall practice patterns, context, order of techniques for managing hemorrhage	•Start conservatively (medications, massage) •Hysterectomy in emergency •Management – patient/situation dependent	They’d start with medications. If that didn’t work, they’d try B-Lynch or Uterine artery ligation. If it’s the patient’s first baby … well actually for everyone … they’d try to conserve the uterus. Then, they’d try a balloon. Then a hysterectomy. If the patient is unstable, they might go to hysterectomy.
		It’s not just the number of children the woman has had. The reality is that hysterectomy comes with other risks too like injury to the bladder and other things. There are a lot of risks. So it’s not just the number of children she’s had [that influences us to do or not do a hysterectomy]. We look at the patient situation and see if the hemorrhage that can be controlled [with other conservative measures].
Success of techniques (B-Lynch and UBT) when performed	•B-Lynch: Avoid hysterectomy •UBT: Effective (in atony) •Both: Controlled hemorrhage	For the patient it went really well, there wasn’t a need for hysterectomy, it went really well, I think it’s an alternative that we have, that we can use if we have the knowledge and know how to do it, because if we don’t do it adequately it won’t work. At least the experience I had was positive, it went well.
**Hospital Resource and Personnel Limitations**		
UBT Challenges: Resource limitations (time, supplies)	•Lack of supplies (Bakri) •Lack of supplies on hand (condom) •Time delay	Not everyone knows how to place [UBT], the majority of us have never done it, and second because there aren’t any. In the labor area where postpartum hemorrhage happens, the condoms and everything to do it are not very available. It takes time to get all the materials. So it’s lost time.
**Decision-Making Hierarchy**		
Attending Decision-Making	•Attending physicians make the decisions •Need to involve attending physicians •Attending physicians unfamiliar	Attendings^b^ in our hospital are not familiar with this suture (B-Lynch). So it’s not something we use because when we find ourselves dealing with an obstetric hemorrhage, usually we call the attending to make a decision together, so when an attending doesn’t have experience doing this type of suture, they don’t feel comfortable doing it with us, who are in training.
		Here [at the conference], as you can see, the residents are getting training. There are only two attendings here, and we’ve done this training before. Each hospital has their own attendings. So part of the issue is that the training needs to be done with the attendings from all the hospitals… they (the attendings) are the ones who have to learn and put [the skills] into practice…It’s not the resident’s responsibility [to make decisions]. It’s our [attending's] responsibility.
Hierarchy and lack of trainee autonomy	•Residents not responsible for decision-making •Need to consult attending/superior	If you’re a medical student or trainee, it’s not your choice. It’s the attending who makes the decision always. If it’s an emergency situation and they think it would help, they would do it. In the situation I saw, I was a resident helping the attending. I observed but the attending placed the [B-Lynch] sutures.
**Challenging Nature of Emergencies**		
Challenging to learn during emergency	•Stressful and difficult to learn in real life •Hard to learn in an emergency	It’s hard to learn in real life when a patient’s life is in danger and there’s such a high level of concern.
Emergency decision-making: Pressure, stress, and fear	•Quick decision-making •Need to be confident	I was alone. I was really stressed out. I didn’t do the best job. [In an emergent situation], my instinct would be to do a hysterectomy because I didn’t have time to wait. I didn’t have access to a blood bank. In reality, I couldn’t think about [uterine sparing measures like a B-Lynch] Again, the fact that [UBT] is not done very often, [the barrier] is fear. It is fear that this technique will not be secure or successful because we don’t have a lot of practice with this [technique]. We have more practice in the OR… I think that the fear is one of the things that prevents us ([which is why] we don’t use [UBT] a lot).
**Impact of Simulation Training, GO MOMS Program**		
Effectiveness of Simulation for Learning: Safe Practice	•Technical aspects of skill •Introduction of new skills	The program is great. The models are really good and help us figure out the technical aspects of these skills.
Positive influence of GO MOMS	•Seen changes in practice since GO M MOMS •B-Lynch more common after GO MOMS introduction	I think the most powerful thing that you have showed us is the B-Lynch. We now are using a lot of more B-Lynch. Before this, we really don’t use it. Never.
Desire for more training and practice	•Need for regular trainings •More practice wanted	We took the course, but we aren’t constantly reinforcing, doing simulation workshops, for example, so I think we are not as comfortable with this method.

^a^Quotes represent the viewpoints of participants who had participated in GO MOMS training at least once prior to 2019.

^b^“Attending” was translated from the Spanish word for “boss” (“jefe”)

The qualitative analysis demonstrated that unfamiliarity and time/resource limitations influenced the ability of providers, especially residents and trainees, to implement new procedures (or new ways of doing existing procedures). For example, UBT was a procedure many were familiar with, but due to limited Bakri balloons, lack of supplies on hand for condom-catheter UBTs, and lack of practice with UBT in general, UBT uptake was a significant challenge. Conversely, B-Lynch was more easily implemented given the comfort in the OR and readiness of supplies.

*Not everyone knows how to place [UBT], the majority of us have never done it, and second because there aren’t any. In the labor area where postpartum hemorrhage happens, the condoms and everything to do it are not very available. It takes time to get all the materials. So it’s lost time.*

Multiple interview respondents noted the importance of attending physicians in decision-making and that decision-making hierarchies impacted procedure implementation. If attending physicians were unfamiliar with a certain procedure, it seemed less likely that residents would be able to implement it.

*Attendings in our hospital are not familiar with this suture (B-Lynch). So it’s not something we use because when we find ourselves dealing with an obstetric hemorrhage, usually we call the attending to make a decision together, so when an attending doesn’t have experience doing this type of suture, they don’t feel comfortable doing it with us, who are in training.*

## Discussion

Our study showed that providers with previous GO MOMS simulation training reported more comfort with B-Lynch for the management of PPH compared to those with no prior training. We also found that providers with previous GO MOMS simulation training reported more comfort with UBT for the management of PPH compared to those with no prior training, although this result was not statistically significant. Qualitative interviews provided insight into the challenges that hinder uptake of UBT with an emphasis on resource limitations and decision-making hierarchies. Utilization of a mixed-methods approach allowed us to evaluate provider attitudes, practice patterns, and patient management for PPH before and after the introduction of SBT in Guatemala. Analysis of multiple-choice knowledge surveys indicated that providers with previous GO MOMS training were significantly more comfortable doing B-Lynch with and without supervision and were also more likely to have taught B-Lynch to someone else compared to those who had not received previous GO MOMS training. There was no difference between groups in terms of comfort with UBT or teaching UBT to others. These findings suggest that SBT is successful for skills-based teaching and can also promote a “ripple effect” of knowledge acquisition and transfer among colleagues, specifically with procedures that are more commonly practiced in the local context, such as B-Lynch.

The qualitative interviews revealed challenges that hinder uptake and implementation of UBT compared to B-Lynch. Several respondents pointed to resource limitations (lack of commercially available uterine balloons), additional time required to assemble supplies for condom-catheter uterine balloon tamponades, and overall hospital culture as factors limiting UBT use. Implementation gaps due to health system barriers have similarly been identified in a mixed-methods study evaluating implementation of PPH guidelines in Uganda [19]. Data from our qualitative interviews also suggested that education of the majority of attending physicians is crucial for implementation and uptake of any new procedures since emergent decisions around patient management are often made at the attending level, and residents feel less comfortable suggesting a procedure that their attendings are unfamiliar with.

Our findings agree with those in the literature that B-Lynch suture and UBT are amenable to simulation training in resource-limited settings [20, 21]. Prior work by us (GO MOMS) [12] demonstrated that SBT was efficacious for use in training in low-resource settings by increasing short and long-term (> 6 month) clinical knowledge and self-efficacy regarding the management of obstetric emergences. In addition, others (PRONTO) have found that SBT can have an impact on local practice patterns, such as reducing the local incidence of cesarean sections [22]. We are able to add to this emerging body of literature by incorporating a mixed-methods approach, examining provider attitudes, local practice patterns, and patient outcomes. This wider view allows a deeper understanding of the challenges that arise when trainees attempt to implement new procedures after participation in a SBT session. Many evaluation studies assessing training programs examine pre- and post-test outcomes alone[13], but without robust evaluation of provider experiences after training, it is impossible to identify the human and structural barriers that may impede the translation of knowledge into implementation.

Potential learning opportunites of SBT that were not analyzed with this study but should be considered for future research in LMICs include the introduction of simulation “drills” to ensure a unifed approach by the entire labor and delivery team during obstetrical emergencies. Checklists, which have emerged as a powerful tool to unify information and task coordination [23] in urgent situations, are best introduced and practiced in a simulated setting. Additionally, the introduction and practice of communciation techniques such as closed loop or call outs are effectively taught in a simulated environment [24].

The strengths of this study are several: first, this study evaluated an educational platform that is easily reproducible in a low-resource setting; second, we incorporated local resources in our didactics and simulation activities which led to a strong collaboration from the local community and medical team; third, this study revealed important barriers to implementation of knowledge. However, this study is not without limitations. With regards to the multiple-choice knowledge survey, participants differed between groups, with more attending physicians in the group that had not previously received GO MOMS training. As a result of this, we are unable to determine if the increased comfort with B-Lynch observed among providers who had received previous GO MOMS training (more resident participants) versus those who did not (more attending physician participants) is attributable to SBT, level of training, or a combination of both. For instance, residents may attend more deliveries than attendings, and therefore may report more comfort with certain techniques. The presence of more residents in the group that had previously received GO MOMS training suggests that the GO MOMS trainings were more targeted towards residents, and residents may have also been more available to attend SBT sessions (compared to attendings). Future recruitment efforts should focus more resources on also involving attendings in SBT, given that they are at the “top” of decision-making hierarchies and their “buy-in” is needed for the successful implementation of new practices. In addition, given that a goal of our SBT program was to provide training that is relevant to providers in their local contexts, our SBT program, and others, can benefit from conducting frequent needs analyses to better understand what resources are (or are not) available among trainees, which would allow for training efforts to be better tailored to fit the local context. For example, our curriculum had already incorporated training around condom-catheter UBTs, given that Bakri balloons are limited in LMICs, and findings from this study suggest that future training efforts may benefit from focusing even more on condom-catheter UBTs. For the qualitative analysis, one possible limitation is the maintenance of objectivity; we attempted to minimize this by involving multiple investigators who participated in various levels of data coding and analysis.

## Conclusions

This study utilized a mixed-methods design to better understand the impact of simulation-based training at several levels that would not adequately be captured with one type of evauation alone. Capturing data across several sources allowed us to gain insight into the mechanisms hindering uptake of procedures taught in simulation, allowing for future adjustments in program development. In our experience, there were also sociocultural factors that enhanced and deepened our results, which would not have been captured through one modality alone. Although this study is specific to procedures used to manage the obstetric emergency of PPH, our findings can be used by the global simulation learning community as a whole. Study designs or program evaluations that utilize a “one-pronged” approach may not be feasible in certain contexts based on numeous barriers that challenge data collection. A mixed-methods study could serve as a useful framework for others who are planning to carry out program evaluations in LMICs.

## Supplementary Information

**Additional file 1.** Multiple Choice Survey English.

**Additional file 2.** Qualitative Interview Guide.

## Data Availability

The datasets used and/or analysed during the current study are available from the corresponding author on reasonable request.

## Abbreviations

SBT: Simulation based training
PPH: Postpartum hemorrhage
UBT: Uterine balloon tamponade
LMICs: Low and middle income countries
