# Supplementary material for: Mixed methods evaluation of simulation-based training for postpartum hemorrhage management in Guatemala
Source: BMC Pregnancy Childbirth. 2022 Jun 24;22:513. doi: 10.1186/s12884-022-04845-2 (PMC9229498; doi:10.1186/s12884-022-04845-2)
Supplement: Supplementary file 1 — Additional file 1. Multiple Choice Survey English. [file 12884_2022_4845_MOESM1_ESM.doc]

**Knowledge Survey – Guatemala GOMOMS 2019**

Hello! We are part of a Stanford research team that has been brought in to help improve the training programs for GOMOMS. This survey is being carried out as part of an evaluation process for GOMOMS. Thank you!

**Demographics:**

1. What is your job title?

- 1 year resident
- 2 year resident
- 3 year resident
- 4 year resident
- Attending physician
- Other
- If “Other,” please specify: _____________

2. If “Attending physician," how many years have you been practicing medicine (since completing medical school)?

- 1-4 years
- 5-10 years
- >10 years

3. How many years have you been working at your hospital?

- 1-4 years
- 5-10 years
- >10 years

4. Have you received simulation training from GOMOMS Stanford in the past?

- Yes
- No

**General practice patterns:**

5. Approximately how many vaginal deliveries do you perform in a typical month?

- 0-10
- 10-30
- >30
- I am not sure

6. Approximately how many C-sections do you perform in a typical month?

- 0-10
- 10-30
- >30
- I am not sure

7. Approximately how many postpartum hemorrhages (>1000cc of blood loss after delivery) do you manage in a typical month?

- 0-5
- 5-10
- >10
- I am not sure

8. Approximately how many spontaneous abortions do you manage in a typical month?

- 0-5
- 5-10
- >10
- I am not sure

**B-Lynch:**

9. Have you ever been taught how to do a B-lynch?

- Yes
- No

10. If so, where did you first learn how to do a B-lynch?

- GO MOMS Simulation course by Stanford
- From one of my hospital program residents or attendings
- Independent study (I read about it)

11. When did you learn this skill? (Year)

- ______

12. Have you taught anyone else to perform a B-lynch?

- Yes
- No

13. How comfortable do you feel performing a B-lynch?

- - - Can do without supervision
    - Can do with supervision
    - Not comfortable

14. How many times have you ever performed a B-lynch?

- 0 times
- 1-5
- 5-10
- I am not sure

15. When you did a B-lynch, how many of them were done at time of C-sections?

- All of them
- Most of them
- Some of them
- Few of them
- None of them

16. When you did a B-Lynch, how many of them were done after a vaginal delivery?

- All of them
- Most of them
- Some of them
- Few of them
- None of them

17. How often after performing a B-lynch did you still have to proceed to hysterectomy to control the bleeding?

- All of them
- Most of them
- Some of them
- Few of them
- None of them

**Uterine balloon tamponade (UBT) or Bakri balloon:**

18. Have you ever been taught how to do a uterine balloon tamponade (UBT)?

- Yes
- No

19. If so, where did you first learn how to do a UBT?

- GO MOMS Simulation course by Stanford
- From one of my hospital program residents or attending
- Independent study (I read about it)

20. When did you learn this skill? (Year)

- __________

21. Have you taught anyone else to perform a UBT?

- Yes
- No

22. How comfortable do you feel with UBT?

- - - Can do without supervision
    - Can do with supervision
    - Not comfortable

23. How many times have you ever performed a UBT?

- 0 times
- 1-5
- 5-10
- I am not sure

24. When you did a UBT, how many of them were done at time of C-sections?

- All of them
- Most of them
- Some of them
- Few of them
- None of them

25. When you did a UBT, how many were done after a vaginal delivery?

- All of them
- Most of them
- Some of them
- Few of them
- None of them

26. How often after performing a UBT, did you still have to proceed to hysterectomy to control the bleeding?

- All of them
- Most of them
- Some of them
- Few of them
- None of them

**Spontaneous Abortion:**

27. How do you typically manage a spontaneous abortion when the patient is bleeding heavily?

- D&C in the Operating Room
- D&C not in the Operating Room (clinic or Emergency Department)
- Medical management with misoprostol
- Expedient management (wait for the patient to complete the abortion on her own)

28. How do you typically manage a spontaneous abortion when the patient is spotting?

- D&C in the Operating Room
- D&C not in the Operating Room (clinic or Emergency Department)
- Medical management with misoprostol
- Expedient management (wait for the patient to complete the abortion on her own)

29. How do you typically manage a spontaneous abortion when the patient is not bleeding?

- D&C in the Operating Room
- D&C not in the Operating Room (clinic or Emergency Department)
- Medical management with misoprostol
- Expedient management (wait for the patient to complete the abortion on her own)

**Dilation & Curettage (D&C):**

31. When performing a D&C, what is your procedure of choice?

- Suction curettage
- Sharp curettage
- Manual vacuum aspiration
- Other
- If “Other,” please specify: _____________

32. If you perform Manual vacuum aspiration (MVA), where were you trained?

- One of my hospital providers taught me
- Independent study (I read about it)

33. If you perform MVA, when did you learn this skill? (Year)

- _____________

34. How comfortable do you feel performing a Manual vacuum aspiration (MVA)?

- Can do without supervision
- Can do with supervision
- Not comfortable

35. Where are MVAs performed?

- Clinic
- Operating Room
- Other
  - If “Other”, please specify: ___________

36. For MVA, what kind of anesthesia is provided?

- ___________

**Medication abortion:**

37. Have you ever been taught how to use medication (misoprostol) for the management of a spontaneous abortion?

- Yes
- No

38. If so, where did you learn how to use medication (misoprostol) for the management of a spontaneous abortion?

- One of my hospital providers taught me
- Independent study (I read about it)

1. When did you learn this skill? (Year)

- ____________

1. How many times have you ever prescribed misoprostol for a spontaneous abortion?

- 0 times
- 1-5
- 5-10
- I am not sure

1. How comfortable do you feel prescribing misoprostol?

- Can do without supervision
- Can do with supervision
- Not comfortable

1. We are also seeking 30 residents to participate in a 15-30 minute interview to help us plan future GOMOMS training programs. Would you be interested in participating in the interview?

- Yes
- No

1. If YES, we will contact you to schedule an interview time. Please include your email or phone number here: ___________________________
