# Supplementary material for: Mixed methods evaluation of simulation-based training for postpartum hemorrhage management in Guatemala
Source: BMC Pregnancy Childbirth. 2022 Jun 24;22:513. doi: 10.1186/s12884-022-04845-2 (PMC9229498; doi:10.1186/s12884-022-04845-2)
Supplement: Supplementary file 2 — Additional file 2. Qualitative Interview Guide. [file 12884_2022_4845_MOESM2_ESM.pdf]

Part II. Qualitative Interview Question Guide

Interviewer: We are part of a research team from Stanford brought in to help improve GOMOMS training programs. This interview is being conducted as part of an evaluation process to help us plan future GOMOMS training programs. We will be interviewing about 30 residents. The interviews will be recorded, transcribed, and compiled for the purposes of research. Nothing you say will ever be identified with you personally, however we would like to link your interview answers to your online survey answers using your phone number. As we go through the interview, if you have any questions about why we are asking you something, please ask us. Also, if there is anything you do not want to answer, please just let us know.

0. Do you provide your verbal consent to participate in the interview? Do you have any questions before we begin?

1. What is your job title?

- 1 year resident
- 2 year resident
- 3 year resident
- 4 year resident
- Attending physician
- Other
    - If "Other," please specify: _____________

2. If "Attending physician," how many years have you been practicing medicine (since completing medical school)?

- 1-4 years
- 5-10 years
- >10 years

3. How many years have you been working at your hospital?

- 1-4 years
- 5-10 years
- >10 years

4. Have you received simulation training from GOMOMS Stanford in the past?

- Yes
- No

2. Now we would like to ask you about your experiences performing different procedures in Obstetrics and Gynecology, starting with B-lynch suture.
2.1 Have you ever performed or observed a Blynch?
<u>If YES,</u>

2.1. What challenges have you encountered in performing B-lynch?

2.1.1. What challenges prevent you from being able to perform B-lynch?
2.1.2. What complications have you witnessed while performing B-lynch?

2.2. What positive experiences have you had with performing B-lynch?

3. Now we would like to ask you about your experiences performing uterine balloon tamponade (UBT).
3.1 Have you ever placed or observed the placement of a UBT?
<u>**If YES,**</u>

- **3.1.** What challenges have you encountered in performing UBT?
  - **3.1.1.** What challenges prevent you from being able to perform UBT?
  - **3.1.2.** What complications have you witnessed while performing UBT?
- **3.2.** What positive experiences have you had with performing UBT?

4. Finally, we would like to talk with you about your experiences managing spontaneous abortions, or miscarriages.

- **4.1.** What do you know about using medication(misoprostol) to manage a spontaneous abortion?
  - **4.1.1.** Who do you believe are the good candidates for misoprostol?

4.1.2. What dose of misoprostol do you prescribe? Do you repeat any dosing?
4.1.3. What is the mode of administration (oral, vaginal)?

- **4.2.** What challenges or complications have you experienced (or heard about) with medical management of spontaneous abortion?
- **4.3.** What positive experiences have you had (or heard about) with medical management of spontaneous abortion?
- **4.4.** If applicable, what hesitations do you have about using medication to manage a spontaneous abortion?
- **4.5.** If applicable, what would you like to learn more about medication abortion?
- **4.6.** Have you seen patients self-manage miscarriage at home?

4.6.1. If so, what have they used to self-manage a miscarriage at home?

5. Thank you so much for your responses. This interview has been very helpful to us. Do you have any other thoughts, feelings, or ideas that you would like to share with us about the course, or anything else to add?
